# Liposomal C6 Ceramide Activates Protein Phosphatase 1 to Inhibit Melanoma Cells

**DOI:** 10.1371/journal.pone.0159849

**Published:** 2016-09-15

**Authors:** Fangzhen Jiang, Kai Jin, Shenyu Huang, Qi Bao, Zheren Shao, Xueqing Hu, Juan Ye

**Affiliations:** 1 Department of Plastic and Reconstructive Surgery, the Second Affiliated Hospital of Zhejiang University, College of Medicine, Hangzhou, 310009, China; 2 Department of Ophthalmology, the Second Affiliated Hospital of Zhejiang University, College of Medicine, Hangzhou, 310009, China; Louisiana State University Health Sciences Center, UNITED STATES

## Abstract

Melanoma is one common skin cancer. In the present study, the potential anti-melanoma activity by a liposomal C6 ceramide was tested *in vitr*o. We showed that the liposomal C6 (ceramide) was cytotoxic and anti-proliferative against a panel of human melanoma cell lines (SK-Mel2, WM-266.4 and A-375 and WM-115). In addition, liposomal C6 induced caspase-dependent apoptotic death in the melanoma cells. Reversely, its cytotoxicity was attenuated by several caspase inhibitors. Intriguingly, liposomal C6 was non-cytotoxic to B10BR mouse melanocytes and primary human melanocytes. Molecularly, liposomal C6 activated protein phosphatase 1 (PP1) to inactivate Akt-mammalian target of rapamycin (mTOR) signaling in melanoma cells. On the other hand, PP1 shRNA knockdown or exogenous expression of constitutively activate Akt1 (CA-Akt1) restored Akt-mTOR activation and significantly attenuated liposomal C6-mediated cytotoxicity and apoptosis in melanoma cells. Our results suggest that liposomal C6 activates PP1 to inhibit melanoma cells.

## 1. Introduction

Melanoma is one common skin cancer [1,2,3,4,5]. It is characterized by rapid disease progression and early invasion/metastasis to other organs [6]. It is estimated that metastatic or recurrent melanoma causes over 8000 deaths each year [5]. In addition, melanoma is resistant to almost all traditional chemotherapy agents [1,2,3,4]. Currently, dacarbazine and temozolomide (TMZ) are routinely prescribed for melanoma chemotherapy. Yet, the response rate is often less 15–20% [1,2,3,4]. Therefore, it is urgent to explore novel and more potent anti-melanoma agents.

Ceramides are a family of lipid molecules that are enriched within cell membranes [7,8]. Ceramides could also function as active signaling molecules [7,8]. Among all the ceramides, the short-chain cell permeable ceramides (C2, C4, C6 and C8) have displayed promising anti-tumor activity, either alone or in combination with traditional anti-cancer agents (reviewed in [9,10,11,12]). C6 ceramide has been tested in melanoma cells, and showed decent *in vitro* cytotoxicity [13]. Yet, the systematic use of the short-chain ceramides is extremely limited due to their poor solubility [14]. Therefore, liposome-based nanotechnology delivery systems have been developed to assist ceramide delivery *in vivo* [14,15,16,17,18]. In the current study, we investigated the potential anti-melanoma activity by a liposomal C6 ceramide [14,18]. The underlying mechanisms were also analyzed.

## 2. Materials and Methods

### 2.1. Chemicals and reagents

Liposomal C6 (ceramide), liposome ghost vehicle and free C6 (ceramide) were provided by Bo Zhang’s Lab at Tianjin Medical University [19]. The caspase-3 specific inhibitor Ac-DEVD-CHO, the caspae-9 specific inhibitor Ac-LEHD-CHO and the pan caspase inhibitor Ac-VAD-CHO were purchased from Peptide Institute (Osaka, Japan). Antibodies of PP1α/β/λ were obtained from Santa Cruz Biotech (Santa Cruz, CA). All other antibodies utilized in the study were purchased form Cell Signaling Tech (Denver, MA). Cell culture reagents were provided by Calbiochem (Shanghai, China).

### 2.2. Cell culture

Melanoma cell lines SK-Mel2, WM-266.4, A-375 and WM-115 were obtained from American Type Culture Collection (ATCC, Manassas, VA). Melanoma cells were maintained in RPMI medium, supplemented with 10% heat-inactive fetal bovine serum (FBS), 1% penicillin–streptomycin and 4 mmol/L L-glutamine, in a CO_2_ incubator at 37°C. B10BR mouse melanocytes, obtained from Shanghai Biological Institute (Shanghai, China), were cultured in Hams F12 supplement with 10 heat-inactivated calf serum (FCS, Gibco), 50 ng/mL phorbol 12-myristate 13-acetate (TPA, Sigma) and 1% penicillin–streptomycin. Primary human melanocytes from neonatal foreskin (Cascade Biologics/Life Technologies, Shanghai, China) were cultured in Medium 254 and Human Melanocyte Growth Supplement (HMGS2; Cascade Biologics/Life Technologies) and propagated for ≤15 population doublings.

### 2.3. Cell survival MTT assay

Standard MTT (3-(4,5-Dimethylthiazol-2-yl)-2,5-diphenyltetrazolium bromide, Sigma) assay was performed in 96-well plates as described previously [18]. Optic density (OD) value at 570 nm of treatment group was normalized as percentage change of that of untreated control group [20].

### 2.4. Caspase-3/-9 activity assay

Following treatment, melanoma cells were examined for the caspase-3/-9 activity via caspase-3/-9 activity kit (Clontech Corporation, Carlsbad, CA) with the manufacturer's protocol [21]. Briefly, 200 μg of the protein lysates were incubated with 200 μM of the caspase-3 substrate DEVD-pNA or 200 μM of the caspase-9 substrate Ac-LEHD-pNA at 37°C for 2 hours. Absorbance of pNA was detected at 405 nm with a microplate reader (PerSeptive Biosystems, Framingham, MA).

### 2.5. Annexin V detection of apoptosis

Cell apoptosis was detected by the Annexin V Apoptosis Detection Kit (BD Biosciences, Shanghai, China). Briefly, after treatment, melanoma cells were resuspended in 1×Binding buffer, Annexin V-FITC and propidium iodide (PI) (5 μg/ml each). After 15 min incubation, cells were acquired by flow cytometry. Both early apoptotic cells (Annexin V positive, PI negative) and late apoptotic cells (Annexin V positive, PI positive) were detected by FACScan, and subsequently analyzed by CellQuest software. Annexin V percentage was recorded.

### 2.6. Single-stranded DNA (ssDNA) ELISA assay of apoptosis

In the process of apoptosis, DNA denature is a characteristic marker. In the present study, denatured ssDNA was detected via a nucleosomal monoclonal antibody in an ELISA format. Detailed protocol was described in other studies [19,21,22,23]. Briefly, melanoma cells (2.5 ×10^4^/well) were seeded onto 96-well plates. After applied treatment, cell apoptosis was analyzed via the ssDNA ELISA kit (Chemicon, Shanghai, China) according to the attached protocol. The OD value was utilized as a quantitative indicator of cell apoptosis.

### 2.7. Western blots

Cells were washed and incubated in cell lysis buffer [20]. Protein samples were separated by SDS-PAGE gel and electro-transferred to PVDF membranes (Bio-Rad), followed by incubation with primary antibodies [18]. Protein bands were visualized using horseradish peroxidase (HRP)-conjugated secondary antibodies (Santa Cruz), and by the enhanced chemiluminescence (ECL) reagents [19]. The x-ray films were scanned, acquired in Adobe Photoshop, and analyzed with NIH Image J software.

### 2.8. Protein phosphatase activity assay

Protein phosphatase activity was determined with the [^32^P] phosphorylase a protocol as previously described [24]. The assay was performed in a 50-μl aliquot that consisted of 50 mM Tris HCl (pH 7.4), 5 mM caffeine, 0.5 mM EGTA, 0.5 mM EDTA, 50 μM β-mercaptoethanol, and 100 ng of aprotinin (protease inhibitor) with or without 2 μg of protein lysates and 500 pmol [^32^P] phosphorylase a [24]. The assay was initiated by adding the cell lysates and was incubated at 30°C for 5 min. Incubation was rapidly stopped by addition of 30 μl of 60% TCA and 20 μl of BSA (50 mg/ml). Tubes were held in ice for 10 min and then centrifuged at 12,000 g for 5 min. After centrifugation, ^32^P radioactivity was counted in 80 μl of clear supernatant in 7 ml of liquid scintillation fluid. protein phosphatase activity was calculated through the same protocol as described [24]. The protein phosphatase activity of liposomal C6 treatment group was normalized to that of untreated control group.

### 2.9. PP1 shRNA knockdown

The pan PP1 shRNA (sc-43545-SH, Santa Cruz) and scramble control shRNA were purchased from Santa Cruz Biotech (Shanghai, China). The PP1 shRNA sequence was described in the previous study [25]. For shRNA transfection, melanoma cells were seeded at 50% confluence. The shRNA vector was introduced by Lipofectamine 2000 (Invitrogen, Carlsbad, CA), according to the manufacturer’s protocol. The stable cells expressing PP1 shRNA were selected by puromycin (2.5 μg/ml) for 2–3 weeks. Western blot assay was always performed to test PP1α expression in stable cells.

### 2.10. Constitutively active-Akt1 (CA-Akt1) transfection and stable cells selection

The constitutively active mutant of Akt1 (CA-Akt1) cDNA sequence was provided by Dr. Teng’s group at Jining Medical University [26,27]. CA-Akt1 was inserted into the pSuper-puro-GFP vector and was transfected via Lipofectamine 2000 reagent (Invitrogen), according to the manufacturer’s protocol. The stable cells were selected by puromycin (2.5 μg/ml) for 2–3 weeks. Western blot assay was performed to test CA-Akt1 in stable cells.

### 2.11. Statistical analysis

The values in the figures were expressed as the means ± standard deviation (SD). Statistical analysis of the data was performed by ANOVA. Values of p < 0.05 were considered as statistically different.

## 3. Results

### 3.1. Liposomal C6 inhibits melanoma cell survival and proliferation

**Fig 1:**
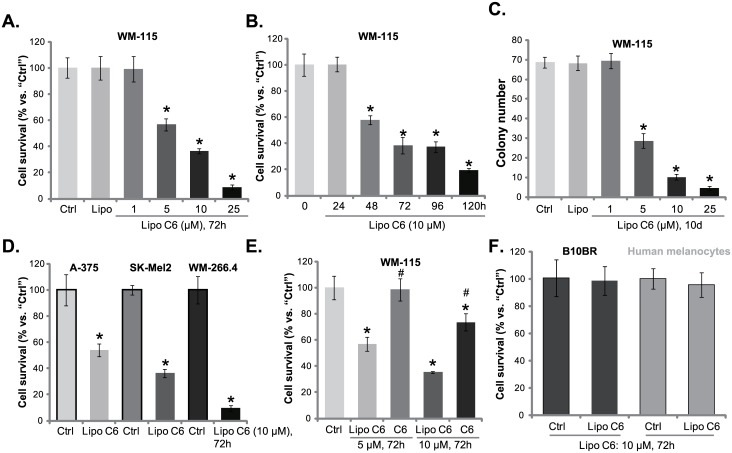
Liposomal C6 inhibits melanoma cell survival and proliferation. Cultured melanoma cell lines (WM-115, SK-Mel2, WM-266.4 and A-375), B10BR mouse melanocytes or primary human melanocytes were either left untreated (“Ctrl”), treated with applied concentrations of liposomal C6 ceramide (“Lipo C6”, A-F), liposomal ghost vehicle (“Lipo”, for A, C), or free C6 ceramide (“C6”, for E), cells were further cultured for applied time, cell survival was tested by MTT assay (A, B, D-F), and cell proliferation was evaluated by colony formation assay (C, for WM-115 cells). Experiments were repeated four times, and similar results were obtained. Data were presented as mean ± SD. * p<0.05 vs. “Ctrl” group. ^#^ p<0.05 vs. “Lipo C6” only group (E).

Cultured WM-115 human melanoma cells were treated with the liposomal C6. MTT assay results in Fig 1A demonstrated that liposomal C6 (at 5–25 μM) dramatically inhibited WM-115 cell survival. Liposomal C6 (10 μM) exhibited a time-dependent effect, the viability OD started to decrease 48 hours after liposomal C6 treatment (Fig 1B). Meanwhile, liposomal C6 treatment dose-dependently inhibited the number of WM-115 colonies (Fig 1C), suggesting its anti-proliferative activity. Note that liposomal ghost (“Lipo”) showed almost no effect on melanoma cell survival nor proliferation (Fig 1A and 1C).

The potential effect of liposomal C6 on other melanoma cell lines was also analyzed. Three other established melanoma cell lines (SK-Mel2, WM-266.4 and A-375) were cultured and treated with liposomal C6 (10 μM). MTT assay results in Fig 1D showed that liposomal C6 was cytotoxic against all these melanoma cell lines. To compare the efficiency between regular free C6 and liposomal C6, WM-115 cells were treated with same concentration (10 μM) of free C6 or liposomal C6, MTT results showed that liposome-packed C6 was significantly more potent than free C6 in suppressing WM-115 cells (Fig 1E). Same results were also obtained in other tested melanoma cell lines (Data not shown). The potential activity of liposomal C6 on normal melanocytes (non-cancerous cells) was tested. MTT results in Fig 1F showed that liposomal C6 (10 μM) failed to inhibit survival of B10BR mouse melanocytes and primary human melanocytes, implying its selective cytotoxicity to cancer cells. Collectively, these results indicate that liposomal C6 exerts cytotoxic and anti-proliferative activity against cultured human melanoma cells.

### 3.2. Liposomal C6 activates apoptosis in melanoma cells

**Fig 2:**
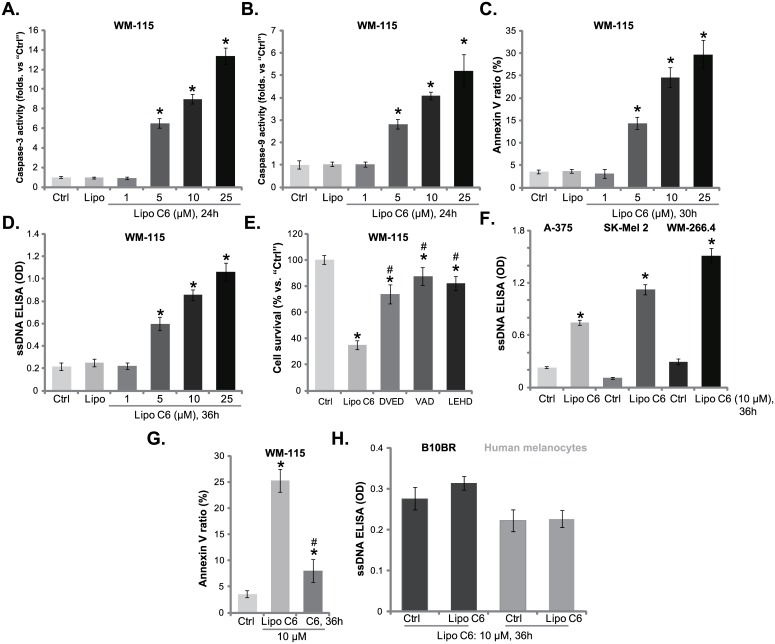
Liposomal C6 activates apoptosis in melanoma cells. Melanoma cell lines (WM-115, SK-Mel2, WM-266.4 and A-375), B10BR mouse melanocytes or primary human melanocytes were either left untreated (“Ctrl”), treated with applied concentration of liposomal C6 ceramide (“Lipo C6”, A-D, F-H), liposomal ghost vehicle (“Lipo”, for A-D), or free C6 ceramide (“C6”, for G), cells were further cultured for applied time, cell apoptosis was analyzed by listed assays (A-D, F-H). WM-115 cells, pre-treated with Ac-DVED-CHO (“DVED”, the caspase-3 inhibitor), Ac-LEHD-CHO (“LEHD”, the caspase-9 inhibitor) or Ac-VAD-CHO (“VAD”, the pan-caspase inhibitor) (50 μM each, 1 hour), were treated with liposomal C6 (10 μM), cells were further cultured for 72 hours, cell survival (E, MTT assay) was analyzed. Experiments were repeated four times, and similar results were obtained. Data were presented as mean ± SD. * p<0.05 vs. “Ctrl” group. ^#^ p<0.05 vs. “Lipo C6” only group (E and G).

Next, we studied the potential effect of liposomal C6 on cell apoptosis. WM-115 cells were treated with indicated concentration of liposomal C6. Results in Fig 2A and 2B showed that liposomal C6 dose-dependently increased activity of caspse-3 and caspase-9 in WM-115 cells. In addition, liposomal C6 (5–25 μM) significantly increased Annexin V percentage (Fig 2C) and ssDNA ELISA OD (Fig 2D). All these results indicated apoptosis activation by liposomal C6 in WM-115 cells (Fig 2A–2D). To study the role of apoptosis in liposomal C6-induced melanoma cytotoxicity, three caspase-based apoptosis inhibitors were applied. Results showed that the caspase-3 specific inhibitor Ac-DEVD-CHO, the caspae-9 specific inhibitor Ac-LEHD-CHO and the pan caspase inhibitor Ac-VAD-CHO dramatically inhibited liposomal C6 (10 μM)-induced WM-115 cell viability reduction (Fig 2E). ssDNA apoptosis ELISA results in Fig 2F confirmed significant apoptosis activation in three other melanoma cell lines after liposomal C6 (10 μM) treatment. Once again, liposomal C6 was more potent than free C6 in inducing apoptosis in WM-115 cells (Fig 2G). Notably, ssDNA ELISA assay results in Fig 2H demonstrated that liposomal C6 failed to induce significant apoptosis in B10BR mouse melanocytes and primary human melanocytes. These results against confirmed its selective activity in cancerous cells. Collectively, liposomal C6 induces caspase-dependent apoptotic death in melanoma cells.

### 3.3. Liposomal C6 activates protein phosphatase, and inhibits Akt-mTOR signaling in melanoma cells

**Fig 3:**
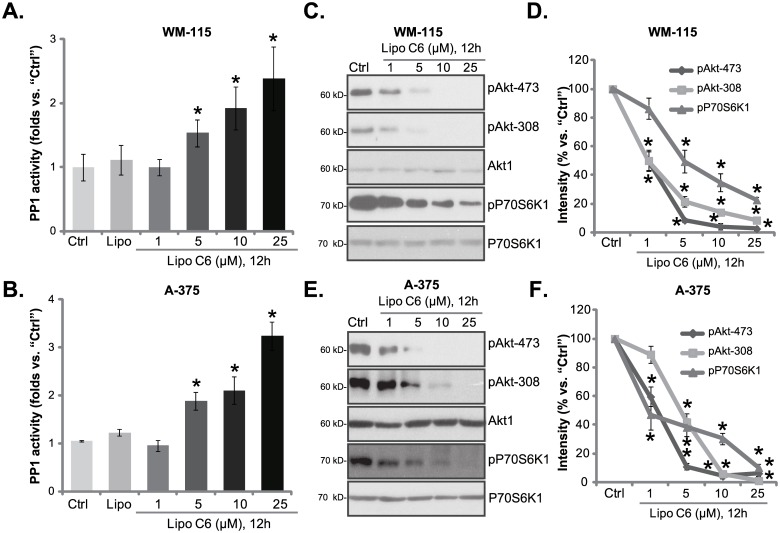
Liposomal C6 activates protein phosphatase, and inhibits Akt-mTOR signaling in melanoma cells. Cultured melanoma cell lines (WM-115 and A-375) were either left untreated (“Ctrl”), treated with applied concentration of liposomal C6 ceramide (“Lipo C6”) or liposomal ghost (“Lipo”), cells were further cultured for applied time, protein phosphatase activity (A and B) and expression of listed kinases (C and E) were tested. Akt and P70S6K1 phosphorylations were quantified (D and F). Experiments were repeated three times, and similar results were obtained. Data were presented as mean ± SD. * p<0.05 vs. “Ctrl” group.

Previous studies have shown that short-chain ceramides could activate the protein phosphatase 1 (PP1) [28,29] and de-phosphorylates Akt to exert cytotoxic or anti-proliferative activity [30]. We thus analyzed protein phosphatase activity in liposomal C6-treated melanoma cells using the method described [24]. Results demonstrated that liposomal C6 dose-dependently increased protein phosphatase activity in both WM-115 (Fig 3A) and A-375 melanoma cells (Fig 3B). As a result, Akt activation was largely inhibited (Fig 3C and 3E). In addition, pP70S6K1, the indicator of mammalian targeted of rapamycin (mTOR) activation, was also inhibited (Fig 3C and 3E). Akt and P70S6K1 phosphorylations in WM-115 and A375 cells were quantified (Fig 3D and 3F). Since, Akt-mTOR activation plays a vital role in melanoma cell survival and proliferation [31], our results suggest that liposomal C6 activates protein phosphatase to inhibit Akt-mTOR signaling and melanoma cell proliferation.

### 3.4. Activation of PP1 is required for liposomal C6-induced anti-melanoma cell activity

**Fig 4:**
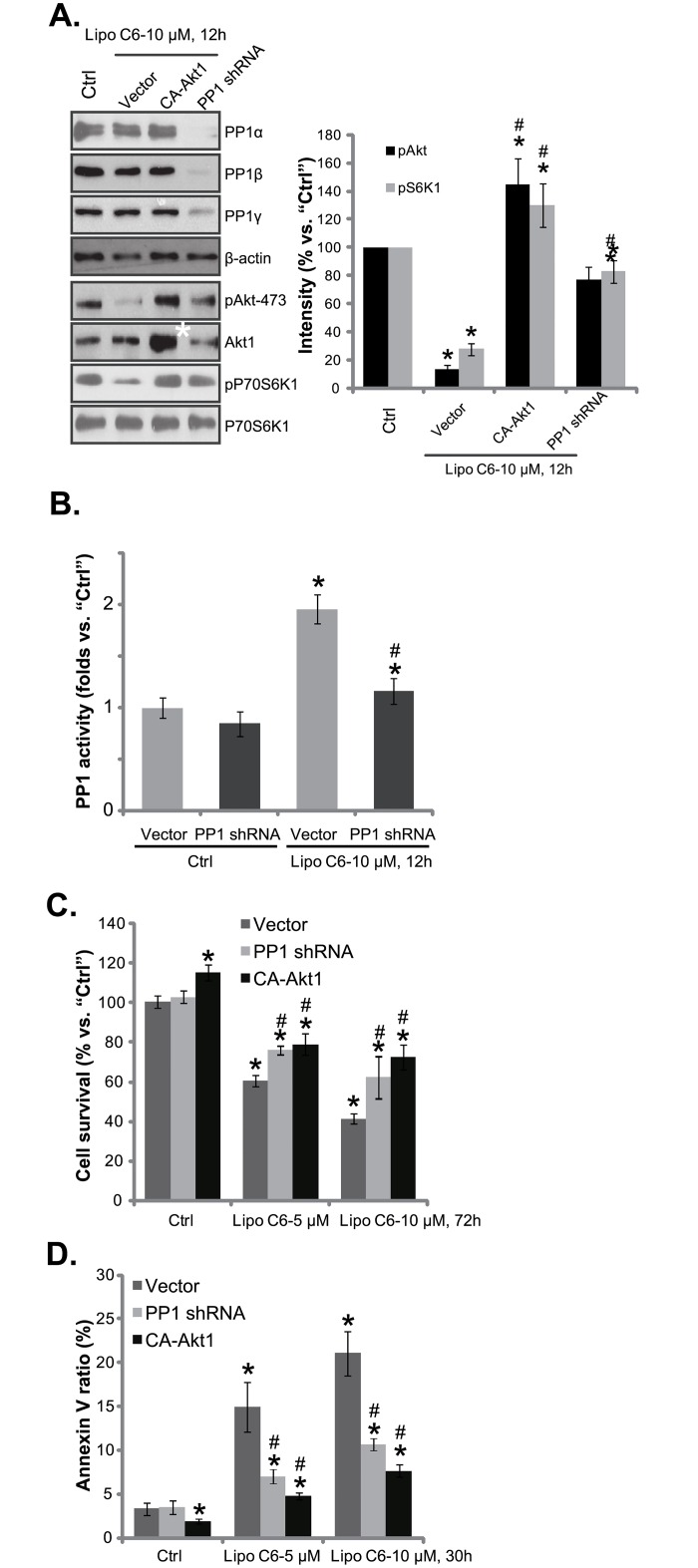
Activation of PP1 is required for liposomal C6-induced anti-melanoma cell activity *in vitro*. Stable WM-115 cells expressing the pan PP1 shRNA, constitutively-activate mutant Akt1 (“CA-Akt1”), or empty vector (“pSuper-puro”) were treated with or without applied concentration of liposomal C6 ceramide (“Lipo C6”), expression of listed proteins was tested by Western blots (A); Relative protein phosphatase activity was shown (B); Cell survival (C), and cell apoptosis (D) were tested by MTT assay and Annexin V assay, respectively. Experiments were repeated three times, and similar results were obtained. Data were presented as mean ± SD. *p<0.05 vs. untreated “Ctrl” group. ^#^ p<0.05 vs. “Lipo C6” of Vector group.

To further support a role of PP1-Akt signaling in liposomal C6-induced anti-melanoma cell activity, we utilized shRNA method to knockdown PP1, and stable WM-115 cells expressing PP1 shRNA were selected. Western blot results in Fig 4A showed that the pan PP1 shRNA significantly downregulated PP1α/β/λ expression in stable WM-115 cells. Consequently, liposomal C6-induced Akt-mTOR inhibition was almost reversed (Fig 4A, also see quantification results in the panel). Significantly, protein phosphatase activity increase by liposome C6 was compromised in PP1-silenced WM-115 cells (Fig 4B). Meanwhile, liposomal C6-induced cytotoxicity (Fig 4C) and apoptosis (Fig 4D) were attenuated in PP1 shRNA-expressing WM-115 cells. These results suggest that liposomal C6 activates PP1 to inhibit Akt-mTOR, causing melanoma cell growth inhibition and apoptosis.

We next introduced a constitutively-active (CA) Akt1 [13] to WM-115 cells. Western blot results in Fig 4A confirmed CA-Akt1 expression (high Akt1 expression, “white star”) in WM-115 cells. CA-Akt1 restored Akt-mTOR activation in liposomal C6-treated WM-115 cells (Fig 4A). More importantly, CA-Akt1-expressing WW-115 cells were resistant to liposomal C6, presenting with significantly reduced cell death (Fig 4C) and apoptosis (Fig 4D). Together, these results indicate that PP1-Akt signaling is required for liposomal C6-induced anti-melanoma cell activity *in vitro*.

## 4. Discussion

Despite the promising anti-cancer activity by the short-chain ceramides [7,10,32], the process of developing these compounds as active pharmaceutical agents has been hampered due to their insolubility [14]. Therefore, liposome-based nanotechnology delivery systems have been developed to assist ceramide delivery *in vivo* [19,33,34,35]. It has been shown that system delivery of liposomal C6 could offer rapid tissue distribution without causing apparent toxicities [15]. In addition, liposomal C6 showed a selective response to cancerous cells [15,18,19]. Recent studies have also concluded that experimental mice were well-tolerated to the liposomal C6 systematic administration [18,19]. In the current study, our *in vitro* studies showed that liposomal C6 (ceramide) exerted potent anti-proliferative and pro-apoptotic activities against a panel of human melanoma cell lines (SK-Mel2, WM-266.4, A-375 and WM-115). Its efficiency was better than free C6 ceramide. Intriguingly, liposomal C6 was non-cytotoxic to B10BR mouse melanocytes and primary human melanocytes.

At the molecular level, we showed that shRNA knockdown of PP1 or introduction of CA-Akt1 alleviated liposomal C6-mediated anti-melanoma activity. These results indicate that PP1-mediated Akt-mTOR inactivation mediated, at least in part, liposomal C6’s cytotoxicity in melanoma cells. However, it should be noted that PP1 shRNA or CA-Akt1 didn’t completely block liposomal C6’ cytotoxicity, indicating that other mechanisms besides the PP1-Akt signaling may also contribute to its actions. As a matter of fact, studies have identified other signaling mechanisms by (liposomal) C6 in various cancer cells, including JNK activation [36], AMP activated protein kinase (AMPK) activation [18,37,38,39,40], growth factor receptor degradation [40] and many others. A recent study by Zhang et al., showed that acute treatment (30 min) of liposomal C6 inhibited melanoma cell migration via phosphorylation of PI3K and PKCζ [41]. Reversely, knockdown or pharmacological inhibition of PKCζ or PI3K restored cancer cell migration following liposomal C6 treatment [41]. It will be interesting to test these signalings in liposomal C6-treated melanoma cells as well.

## 5. Conclusions

Metastatic and recurrent melanoma is still a great challenge to treat [6,42,43]. Stage III melanoma patients are often treated adjuvantly with interferon (IFN)-α, yet its response is far from satisfactory. The metastatic melanoma patients (stage IV) have a median survival of 6–10 months even with current treatments, and the 5-year survival is less than 5% [6,42,43]. Therefore, alternative treatment agents are urgently needed [6,42,43]. Our results show that liposomal C6 potently inhibits melanoma cells *in vitro*. Therefore, the liposomal C6 could be further studied for possible treatment of melanoma.

## References

[1] Hutchinson L (2015) Skin cancer. Golden age of melanoma therapy. Nat Rev Clin Oncol 12: 1 10.1038/nrclinonc.2014.219 25511188

[2] Webster RM, Mentzer SE (2014) The malignant melanoma landscape. Nat Rev Drug Discov 13: 491–492. 10.1038/nrd4326 24981356

[3] Schadendorf D, Hauschild A (2014) Melanoma in 2013: Melanoma—the run of success continues. Nat Rev Clin Oncol 11: 75–76. 10.1038/nrclinonc.2013.246 24419300

[4] Kingwell K (2014) Anticancer drugs: A new weapon against metastatic melanoma. Nat Rev Drug Discov 13: 334. 10.1038/nrd4314 24781544

[5] Siegel R, Ma J, Zou Z, Jemal A (2014) Cancer statistics, 2014. CA Cancer J Clin 64: 9–29. 10.3322/caac.21208 24399786

[6] Braeuer RR, Watson IR, Wu CJ, Mobley AK, Kamiya T, Shoshan E, et al (2014) Why is melanoma so metastatic? Pigment Cell Melanoma Res 27: 19–36. 10.1111/pcmr.12172 24106873

[7] Henry B, Moller C, Dimanche-Boitrel MT, Gulbins E, Becker KA (2011) Targeting the ceramide system in cancer. Cancer Lett. 10.1016/j.canlet.2011.07.010 21862212

[8] Dimanche-Boitrel MT, Rebillard A, Gulbins E (2011) Ceramide in chemotherapy of tumors. Recent Pat Anticancer Drug Discov 6: 284–293. 10.2174/157489211796957838 21762073

[9] Radin NS (2003) Killing tumours by ceramide-induced apoptosis: a critique of available drugs. Biochem J 371: 243–256. 12558497 10.1042/BJ20021878 PMC1223313

[10] Morad SA, Cabot MC (2013) Ceramide-orchestrated signalling in cancer cells. Nat Rev Cancer 13: 51–65. 10.1038/nrc3398 23235911

[11] Mullen TD, Obeid LM (2012) Ceramide and apoptosis: exploring the enigmatic connections between sphingolipid metabolism and programmed cell death. Anticancer Agents Med Chem 12: 340–363. 21707511 10.2174/187152012800228661

[12] Mullen TD, Obeid LM (2011) Ceramide and Apoptosis: Exploring the Enigmatic Connections Between Sphingolipid Metabolism and Programmed Cell Death. Anticancer Agents Med Chem. 10.2174/187152012800228661 21707511

[13] Yu T, Li J, Sun H (2010) C6 ceramide potentiates curcumin-induced cell death and apoptosis in melanoma cell lines in vitro. Cancer Chemother Pharmacol 66: 999–1003. 10.1007/s00280-010-1374-1 20521051

[14] Kester M, Bassler J, Fox TE, Carter CJ, Davidson JA, Parette MR (2015) Preclinical development of a C6-ceramide NanoLiposome, a novel sphingolipid therapeutic. Biol Chem 396: 737–747. 10.1515/hsz-2015-0129 25838296

[15] Zolnik BS, Stern ST, Kaiser JM, Heakal Y, Clogston JD, Kester M, et al (2008) Rapid distribution of liposomal short-chain ceramide in vitro and in vivo. Drug Metab Dispos 36: 1709–1715. 10.1124/dmd.107.019679 18490436

[16] Tran MA, Smith CD, Kester M, Robertson GP (2008) Combining nanoliposomal ceramide with sorafenib synergistically inhibits melanoma and breast cancer cell survival to decrease tumor development. Clin Cancer Res 14: 3571–3581. 10.1158/1078-0432.CCR-07-4881 18519791

[17] Stover TC, Sharma A, Robertson GP, Kester M (2005) Systemic delivery of liposomal short-chain ceramide limits solid tumor growth in murine models of breast adenocarcinoma. Clin Cancer Res 11: 3465–3474. 15867249 10.1158/1078-0432.CCR-04-1770

[18] Chen MB, Jiang Q, Liu YY, Zhang Y, He BS, Wei MX, et al (2015) C6 ceramide dramatically increases vincristine sensitivity both in vivo and in vitro, involving AMP-activated protein kinase-p53 signaling. Carcinogenesis 36: 1061–1070. 10.1093/carcin/bgv094 26116623

[19] Zhai L, Sun N, Han Z, Jin HC, Zhang B (2015) Liposomal short-chain C6 ceramide induces potent anti-osteosarcoma activity in vitro and in vivo. Biochem Biophys Res Commun 468: 274–280. 10.1016/j.bbrc.2015.10.113 26505795

[20] Hu X, Jiang F, Bao Q, Qian H, Fang Q, Shao Z (2015) Compound 13, an alpha1-selective small molecule activator of AMPK, potently inhibits melanoma cell proliferation. Tumour Biol. 10.1007/s13277-015-3854-8 26271666

[21] Banerjee HN, Blackshear M, Williams J, Hawkins Z, Sawyer C, Manglik V, et al (2012) C-6 Ceramide Induces p53 Dependent Apoptosis in Human Astrocytoma Grade4 (Glioblastoma Multiforme) Cells. J Cancer Sci Ther 4: 12 24319543 PMC3849708

[22] Ullal AJ, Marion TN, Pisetsky DS (2014) The role of antigen specificity in the binding of murine monoclonal anti-DNA antibodies to microparticles from apoptotic cells. Clin Immunol 154: 178–187. 10.1016/j.clim.2014.05.007 24873886 PMC4440675

[23] Wu L, Zhang J, Wu H, Han E (2015) DNA-PKcs interference sensitizes colorectal cancer cells to a mTOR kinase inhibitor WAY-600. Biochem Biophys Res Commun 466: 547–553. 10.1016/j.bbrc.2015.09.068 26381179

[24] Gupta RC, Mishra S, Rastogi S, Imai M, Habib O, Sabbah HN (2003) Cardiac SR-coupled PP1 activity and expression are increased and inhibitor 1 protein expression is decreased in failing hearts. Am J Physiol Heart Circ Physiol 285: H2373–2381. 14613911 10.1152/ajpheart.00442.2003

[25] Ilinykh PA, Tigabu B, Ivanov A, Ammosova T, Obukhov Y, Garron T, et al (2014) Role of protein phosphatase 1 in dephosphorylation of Ebola virus VP30 protein and its targeting for the inhibition of viral transcription. J Biol Chem 289: 22723–22738. 10.1074/jbc.M114.575050 24936058 PMC4132779

[26] Sun H, Yu T, Li J (2011) Co-administration of perifosine with paclitaxel synergistically induces apoptosis in ovarian cancer cells: more than just AKT inhibition. Cancer Lett 310: 118–128. 10.1016/j.canlet.2011.06.010 21775054

[27] Shen J, Hong Y, Zhao Q, Zhang JL (2015) Preclinical evaluation of perifosine as a potential promising anti-rhabdomyosarcoma agent. Tumour Biol. 10.1007/s13277-015-3740-4 26269112

[28] Chalfant CE, Rathman K, Pinkerman RL, Wood RE, Obeid LM, Ogretmen B, et al (2002) De novo ceramide regulates the alternative splicing of caspase 9 and Bcl-x in A549 lung adenocarcinoma cells. Dependence on protein phosphatase-1. J Biol Chem 277: 12587–12595. 11801602 10.1074/jbc.M112010200

[29] Chalfant CE, Ogretmen B, Galadari S, Kroesen BJ, Pettus BJ, Hannun YA (2001) FAS activation induces dephosphorylation of SR proteins; dependence on the de novo generation of ceramide and activation of protein phosphatase 1. J Biol Chem 276: 44848–44855. 11502750 10.1074/jbc.M106291200

[30] Yao C, Wu S, Li D, Ding H, Wang Z, Yang Y, et al (2012) Co-administration phenoxodiol with doxorubicin synergistically inhibit the activity of sphingosine kinase-1 (SphK1), a potential oncogene of osteosarcoma, to suppress osteosarcoma cell growth both in vivo and in vitro. Mol Oncol 6: 392–404. 10.1016/j.molonc.2012.04.002 22583777 PMC5528355

[31] Populo H, Soares P, Lopes JM (2012) Insights into melanoma: targeting the mTOR pathway for therapeutics. Expert Opin Ther Targets 16: 689–705. 10.1517/14728222.2012.691472 22620498

[32] Ogretmen B, Hannun YA (2004) Biologically active sphingolipids in cancer pathogenesis and treatment. Nat Rev Cancer 4: 604–616. 15286740 10.1038/nrc1411

[33] Tagaram HR, Divittore NA, Barth BM, Kaiser JM, Avella D, Kimchi ET, et al (2011) Nanoliposomal ceramide prevents in vivo growth of hepatocellular carcinoma. Gut 60: 695–701. 10.1136/gut.2010.216671 21193455

[34] Adiseshaiah PP, Clogston JD, McLeland CB, Rodriguez J, Potter TM, Neun BW, et al (2013) Synergistic combination therapy with nanoliposomal C6-ceramide and vinblastine is associated with autophagy dysfunction in hepatocarcinoma and colorectal cancer models. Cancer Lett 337: 254–265. 10.1016/j.canlet.2013.04.034 23664889 PMC3722309

[35] Ryland LK, Doshi UA, Shanmugavelandy SS, Fox TE, Aliaga C, Broeg K, et al (2013) C6-ceramide nanoliposomes target the Warburg effect in chronic lymphocytic leukemia. PLoS One 8: e84648 10.1371/journal.pone.0084648 24367685 PMC3868606

[36] Verheij M, Bose R, Lin XH, Yao B, Jarvis WD, Grant S, et al (1996) Requirement for ceramide-initiated SAPK/JNK signalling in stress-induced apoptosis. Nature 380: 75–79. 8598911 10.1038/380075a0

[37] Huo HZ, Wang B, Qin J, Guo SY, Liu WY, Gu Y (2013) AMP-activated protein kinase (AMPK)/Ulk1-dependent autophagic pathway contributes to C6 ceramide-induced cytotoxic effects in cultured colorectal cancer HT-29 cells. Mol Cell Biochem 378: 171–181. 10.1007/s11010-013-1608-8 23508272

[38] Chen MB, Zhang Y, Wei MX, Shen W, Wu XY, Yao C, et al (2013) Activation of AMP-activated protein kinase (AMPK) mediates plumbagin-induced apoptosis and growth inhibition in cultured human colon cancer cells. Cell Signal 25: 1993–2002. 10.1016/j.cellsig.2013.05.026 23712032

[39] Ji C, Yang B, Yang YL, He SH, Miao DS, He L, et al (2010) Exogenous cell-permeable C6 ceramide sensitizes multiple cancer cell lines to Doxorubicin-induced apoptosis by promoting AMPK activation and mTORC1 inhibition. Oncogene 29: 6557–6568. 10.1038/onc.2010.379 20802518

[40] Yang L, Zheng LY, Tian Y, Zhang ZQ, Dong WL, Wang XF, et al (2015) C6 ceramide dramatically enhances docetaxel-induced growth inhibition and apoptosis in cultured breast cancer cells: a mechanism study. Exp Cell Res 332: 47–59. 10.1016/j.yexcr.2014.12.017 25576381

[41] Zhang P, Fu C, Hu Y, Dong C, Song Y, Song E (2015) C6-ceramide nanoliposome suppresses tumor metastasis by eliciting PI3K and PKCzeta tumor-suppressive activities and regulating integrin affinity modulation. Sci Rep 5: 9275 10.1038/srep09275 25792190 PMC4366857

[42] Hutchinson L (2014) Skin cancer: less is as good as more in refractory melanoma. Nat Rev Clin Oncol 11: 502. 10.1038/nrclinonc.2014.132 25091612

[43] Eggermont AM, Robert C (2014) Melanoma: smart therapeutic strategies in immuno-oncology. Nat Rev Clin Oncol 11: 181–182. 10.1038/nrclinonc.2014.36 24590131

